# Crosstalk between Red Blood Cells and the Immune System and Its Impact on Atherosclerosis

**DOI:** 10.1155/2015/616834

**Published:** 2015-02-04

**Authors:** Brigitta Buttari, Elisabetta Profumo, Rachele Riganò

**Affiliations:** Department of Infectious, Parasitic and Immune-Mediated Diseases, Istituto Superiore di Sanità, Viale Regina Elena 299, 00161 Rome, Italy

## Abstract

Atherosclerosis is a chronic multifactorial disease of the arterial wall characterized by inflammation, oxidative stress, and immune system activation. Evidence exists on a pathogenic role of oxidized red blood cells (RBCs) accumulated in the lesion after intraplaque hemorrhage. This review reports current knowledge on the impact of oxidative stress in RBC modifications with the surface appearance of senescent signals characterized by reduced expression of CD47 and glycophorin A and higher externalization of phosphatidylserine. The review summarizes findings indicating that oxidized, senescent, or stored RBCs, due to surface antigen modification and release of prooxidant and proinflammatory molecules, exert an impaired modulatory activity on innate and adaptive immune cells and how this activity contributes to atherosclerotic disease. In particular RBCs from patients with atherosclerosis, unlike those from healthy subjects, fail to control lipopolysaccharide-induced DC maturation and T lymphocyte apoptosis. Stored RBCs, accompanied by shedding of extracellular vesicles, stimulate peripheral blood mononuclear cells to release proinflammatory cytokines, augment mitogen-driven T cell proliferation, and polarize macrophages toward the proinflammatory M1 activation pathway. Collectively, literature data suggest that the crosstalk between RBCs with immune cells represents a novel mechanism by which oxidative stress can contribute to atherosclerotic disease progression and may be exploited for therapeutic interventions.

## 1. Introduction

Atherosclerosis is a chronic inflammatory disease of the arterial wall and the leading cause of mortality in the western world [1]. Research over recent years has delineated atherosclerosis as a multifactorial disease because several factors such as hyperlipidemia, hypertension, diabetes mellitus, male gender, obesity, and family history of cardiovascular disease are implicated. Many of these factors promote mechanisms of inflammation and oxidative stress, which are both major characteristics of atherosclerosis which starts as a “response to injury” favoring immune system activation and endothelial dysfunction [1, 2]. Immune system, with elements of both innate and adaptive immunity, contributes positively and negatively to the development of complex atherosclerotic plaques. Plaque macrophages, dendritic cells (DCs), and activated T and B lymphocytes represent the majority of leukocytes in the atherosclerotic lesions and their secretory activity, including proinflammatory cytokine and matrix-degrading protease release, may be related to plaque rupture [3–5]. In particular, immune cells of both innate and adaptive immunity may be activated by various endogenous molecules that have undergone chemical and/or structural modification following oxidative or glycation processes. In this way the immune system activation gives rise to low level inflammation leading to the slow development of atherosclerotic disease [6].

Intraplaque hemorrhage, a common event in vulnerable atherosclerotic lesions, results in the deposition of red blood cells (RBCs) and release of haemoglobin (Hb) [7]. The deposition of RBC membranes within atherosclerotic plaque, providing a large amount of lipids, represents a critical event in the plaque instability [8]. Several lines of evidence have led to attention to a possible pathogenic role of oxidized RBCs in atherogenesis, hypertension [9], coronary artery disease [10], and stroke [11, 12]. The crosstalk of RBCs with immune system has been investigated in the field of basic physiological processes [1, 2, 13–15]. We have recently provided evidence that oxidized RBCs present different immunomodulatory activities and suggested that these activities may contribute to perpetuation of inflammation in the pathogenesis of atherosclerosis [16, 17].

**Figure 1:**
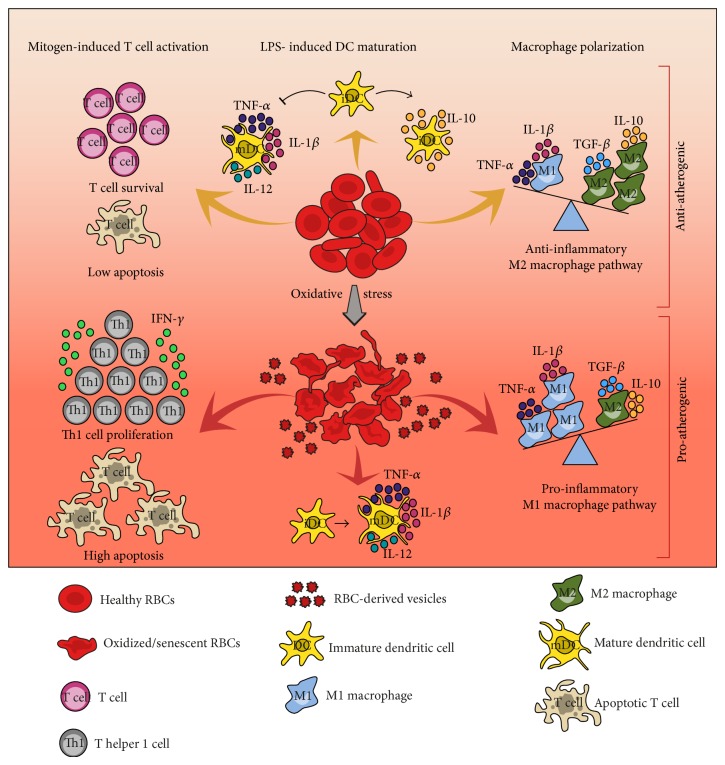
Proposed schematic model showing how the crosstalk between healthy or oxidized/senescent red blood cells (RBCs) and immune cells may exert anti- and proatherogenic effects, respectively. Human healthy RBCs release protein factors with the capacity to sustain T cell survival and to inhibit activation-induced T cell apoptosis. Healthy RBCs are also able to prevent a complete DC maturation in response to LPS, thus inducing phenotypic and functional characteristics typical of immature/tolerogenic DCs characterized by high IL-10 production. Healthy RBCs under noninflammatory conditions might promote a shift toward the anti-inflammatory M2 macrophage pathway. Thus, healthy RBCs may exert an immunomodulatory activity sustaining anti-inflammatory and antiatherogenic mechanisms. Under oxidative stress or store banking conditions, RBCs acquire an oxidized/senescent phenotype resulting in surface antigen modification or release of extracellular vesicles. In this way, oxidized/senescent RBCs or RBC-derived vesicles are capable of augment mitogen-driven T cell proliferation and apoptosis and determine a T helper 1 (Th1) proinflammatory and proatherogenic cytokine response. Oxidized/senescent RBCs, failing to control LPS-induced DC maturation, promote DC maturation toward a DC profile capable of inciting a proinflammatory Th1 cell response. Stored RBCs might polarize macrophages toward the classical M1 macrophage activation pathway associated with proinflammatory cytokine production. We propose that the oxidative injuries on RBCs likely represent dangerous signals for innate and adaptive immune cells, thus contributing or even triggering a damaging process worsening atherosclerotic disease.

In this review, we summarize evidence on the involvement of oxidized RBCs in atherosclerotic disease and in particular on the crosstalk of these cells with innate and adaptive immunity, process that is not traditionally associated with the pathogenesis of this disease (Figure 1).

## 2. Red Blood Cells from Scavenger to Prooxidant Cells

Under physiological conditions, RBCs act as circulating scavengers of oxygen and nitrogen reactive species generated in the vasculature through their well-equipped antioxidant machinery such as reduced glutathione, thioredoxin, ascorbic acid, and vitamin E [18]. When RBCs cross a tissue where an intense production of prooxidant reactive species takes place, the RBC defenses get overwhelmed or are unable to counteract the new prooxidant status of the microenvironment and RBCs become themselves a source of reactive oxygen species (ROS) [18].

Oxidative stress causes a plethora of RBC changes among which are cytoskeleton rearrangement and oxidation and loss of lipid asymmetry. These cells become more rigid and, thus, undergo lysis more easily releasing cytotoxic species in the vasculature. Oxidized RBCs release Hb, heme-Fe, and iron [19, 20]. These molecules are powerful oxidants and sources of radicals and are able to activate in a proinflammatory way endothelial and innate immune cells [21–25], thus contributing to atherosclerotic plaque instability [24, 26]. Moreover, RBCs, exposed to both endogenous and exogenous sources of ROS, undergo not only mechanical alteration such as decreased deformability but also accumulation of oxidative damage products such as lipid oxidation products [27] and 4-hydroxynonenal [28] that may represent endogenously formed factors capable of triggering vascular inflammation [29].

To the current knowledge, ROS represent an important factor into the generation of powerful senescent signals on RBCs. It should be noted that oxidized RBC is not a synonymous of senescent RBC. RBC oxidation may be involved in RBC aging and oxidized RBCs show the phenotype of senescence. The major feature of the senescent RBCs is the clustering and/or the breakdown of Band 3 including the binding of oxidized Hb to high affinity sites on Band 3 [30, 31], and the complexation of Hb with spectrin is also a prominent and probably prior marker of* in vivo* RBC aging process, tightly correlated with increased RBC rigidity, decreased deformability, echinocytosis, and erythrophagocytosis [32, 33]. Considering that Band 3 modifications and modification of preexisting molecule generating pathways are mostly the downward consequence of oxidative provocations, it is likely that in conditions of distorted homeostasis oxidized RBCs result as product of an “accelerating senescence” pathology associated with the premature appearance of cellular senescence phenotype in younger RBCs.

Regarding other RBC signaling pathways, the loss of glycophorin A [34], the externalization of phosphatidylserine (PS) [35], and the reduction of the “marker of self” integrin-associated protein CD47 expression [36] have been documented in circulating senescent RBCs.

A similar senescence phenotype has been reported for RBCs during the storage period [37, 38], with the exception of PS that has not been detected at the external of stored RBC membrane [39]. Refrigerated storage of RBC units for transfusion results in a complex array of physicochemical changes to RBCs, referred to as the storage lesion [40, 41]. A number of the storage-related changes are similar to those seen in senescent or damaged RBCs* in vivo* and may reduce the survival of RBCs when transfused.

Oxidative modifications of RBCs have been reported in several chronic inflammatory diseases such as atherosclerosis, coronary artery disease, metabolic syndrome, and multiple sclerosis [10, 16, 17, 42, 43].

Our research group found alterations in the redox and aging markers of RBCs from patients with carotid atherosclerosis [16, 17]. Patient RBCs exhibited a senescent phenotype and presented an increase of intracellular reactive oxygen and nitrogen species and a decrease of intracellular reduced thiols with respect to RBCs from healthy donors. Of note, our results highlight an increased appearance of oxidized RBCs in the group of male patients in line with the well-known gender differences detected in cardiovascular disease onset and progression [44, 45]. Our experimental evidence on the senescent phenotype acquired by RBCs from healthy subjects following* in vitro* oxidation suggests that in patients with carotid atherosclerosis the appearance of redox and aging markers of RBCs may be ascribed to the high oxidative stress associated with the pathology [17]. High levels of oxidative stress in patients may be generated by atherosclerotic risk factors such as diabetes, smoking, and hypercholesterolemia.

Under oxidative stress or poor glycemic control, RBCs may undergo posttranslational modifications due to nonenzymatic glycosylation (glycation) reaction, a process that leads to the formation of glycated proteins termed advanced glycation end products (AGEs) [46, 47]. The interaction of AGEs with their receptor on endothelial cells or immune cells [48–51] may contribute to vascular perturbation in chronic disorders related to endothelial cell dysfunction such as diabetes and atherosclerosis [52].

Of interest, mouse RBC treated* in vitro* with the antioxidant N-acetyl-L-cysteine (NAC) and then intravenously injected into the sibling mice have showed a prolonged half-life with respect to untreated RBCs [53]. There is also evidence that RBCs stored under the antioxidant and membrane-stabilizing effect of mannitol exhibit a different expression pattern of senescence markers [54]. This evidence supports the hypothesis that RBC oxidation is involved in RBC senescence and complements the findings on the increased appearance of oxidized RBCs in cardiovascular diseases.

Remarkably, oxidized/senescent RBCs have been proposed as useful biomarkers to monitor oxidative alterations in the progression of chronic or acute diseases [18, 55–57].

## 3. Crosstalk between Red Blood Cells and Innate and Adaptive Immune Cells

### 3.1. Crosstalk between Red Blood Cells and Dendritic Cells

Several studies have implicated the role of DCs in progression and destabilization of the atherosclerotic plaque [58–61]. Dendritic cells are key sentinel cells of the innate immune system that possess the ability to stimulate the adaptive immunity [62, 63]. During this process, activated DCs undergo distinct changes in phenotype and function, termed DC maturation [62, 64]. This process involves a redistribution of major histocompatibility complex molecules, an increase in the surface expression of costimulatory molecules, morphological changes, secretion of chemokines, cytokines, and proteases, and surface expression of adhesion molecules and chemokine receptors. A variety of factors can induce DC maturation toward a DC profile capable of inciting primary T cell responses, including lipopolysaccharide (LPS), inflammatory cytokines, ligation of selected cell surface receptors, and viral products [62]. Conversely, several growth factors and cytokines including IL-10 modulate DC maturation, thus favouring the differentiation of tolerogenic DCs, DCs devoted to the maintenance of immunologic tolerance [62].

Schäkel and colleagues [2] have shown* in vitro* that an excess of RBCs, mimicking the physiological conditions in the blood, completely prevents the phenotypical maturation of the proinflammatory subset of circulating DCs (6-sulfo LacNAc+ DCs). The same study shows that RBCs are able to control interleukin (IL)-12 and tumor necrosis factor-alpha (TNF-*α*) production of circulating DCs in response to LPS. The expression of CD47 on RBCs and that of inhibitory receptor signal regulatory protein alpha on circulating DCs appear to be critical for this inhibition [2].

Starting from the concept that* in vitro* senescent or oxidized or eryptotic RBCs modulate surface antigens [65, 66] and that these changes may significantly affect homotypic (RBC-RBC) and heterotypic (e.g., RBC-endothelial) interactions, thus altering RBC functional features [67], our research group has investigated whether RBCs from patients with carotid atherosclerosis maintain their immunomodulatory activity on human monocyte-derived DCs [16]. In this study, we confirmed that human healthy RBCs are able to prevent a complete DC maturation in response to LPS, thus inducing phenotypic and functional characteristics typical of tolerogenic DCs and characterized by reduced CD83, human leukocyte antigen-DR (HLA-DR), CD80, and CD86 surface expression associated with low IL-12, IL-6, and TNF-*α* and high IL-10 production. In the same study, we also provided the first evidence that RBCs from patients with carotid atherosclerosis, unlike those from healthy subjects, fail to control LPS-induced DC maturation. LPS-stimulated DC cultured in the presence of patient RBCs presented a fully mature phenotype characterized by the upregulation of the surface molecules CD83, CD80, HLA-DR, and CD86 and by production of higher levels of proinflammatory cytokines. These results indicate that RBCs from patients with carotid atherosclerosis have an impaired immunomodulatory activity on DC functions, most likely due to the oxidized/senescent RBC phenotype [16]. The altered expression of CD47 at erythrocyte surface or its loss due to vesiculation could represent the main mechanism determining the functional impairment of patient erythrocytes in their crosstalk with DCs.

### 3.2. Crosstalk between Red Blood Cells and Macrophages

In the microenvironment of atherosclerotic plaques, the macrophage polarization state may accelerate or decelerate atherosclerotic disease progression, via activation or attenuation of inflammatory responses. However, the mechanisms underlying macrophage polarization and activation within the plaque remain unclear [68, 69]. The interactions between macrophages and RBCs are important for RBC clearance and homeostasis [57]. In the liver and in the spleen, residential macrophages scrutinize passing RBCs and remove from the circulation those that are at the end of their lifespan or have sustained damage beyond repair [57]. At present there is no consensus as to how macrophages determine which RBCs need to be cleared and which ones can be repaired and/or maintained. Taking into account that RBCs are unable to synthesize new proteins, all “removal” markers must derive from modifications in preexisting molecules or to the acquisition of plasma-derived opsonins directed against these modifications. Furthermore, new evidence suggests that experimental aging of RBC induces a conformational change in CD47 that switches the molecule from an inhibitory into an activating one [70]. Although RBCs do not undergo classical apoptosis since they do not contain a nucleus, mitochondria, or other cellular organelles, the process they undergo has already been termed “eryptosis” since it exhibits many similarities with programmed cell death [71].

After intraplaque hemorrhage, a bulk of RBCs is deposited and degraded over days by macrophage phagocytosis activity through PS-receptors or natural occurring antibodies toward Band 3 or CD47-SIRP*α* interaction [72–75] as a part of a defence mechanism against oxidative burden, and this event promotes atherosclerotic lesion instability [7, 76]. It is highly likely that phagocytosis of senescent or eryptotic RBCs in homeostatic conditions is noninflammatory [77]. The effect of RBC phagocytosis on cytokine secretion in inflammatory condition remains to be elucidated. Another point which needs to be addressed is whether macrophages participate in the resolution of plaque angiogenesis (that likely was the cause of hemorrhage) or actively contribute to it [78].

Yazdanbakhsh et al. [79] have proposed an interesting hypothetical model to explain the effect of stored versus fresh blood transfusion on macrophage plasticity. In this study they analyzed the RBC effects as response to Hb and the Hb breakdown product heme on macrophages. Macrophage plasticity is viewed as a spectrum of activation states ranging from the classic proinflammatory (M1) state, which is induced by the Th1 cytokine interferon- (IFN-) *γ* and bacterial components such as LPS, and the alternatively activated (M2) state, which is associated with the resolution phase of inflammation and driven by IL-4, IL-10, transforming growth factor beta, and glucocorticoids [80]. In a murine RBC storage and transfusion model, Hod et al. [81] showed that the transfusion of RBCs after prolonged storage induced macrophages to polarize toward the classical M1 macrophage activation pathway associated with bactericidal activity and proinflammatory cytokine production conducive of immunostimulation. The transfusion of fresh blood under noninflammatory conditions is associated with less RBC clearance [81] and therefore less loading of macrophages with heme, as well as with upregulation of heme oxygenase and a shift toward the M2 differentiation pathway, which is associated with immunoregulation through the induction of regulatory T cells [79].

During the lifespan of RBCs a loss of haemoglobin and membrane parts occurs from intact RBCs* in vivo*. In a rat model it has been shown that RBC-derived vesicles are rapidly removed from the circulation mainly by liver Kupffer cells and to a lesser extent by other macrophages of the mononuclear phagocyte system through scavenger receptors [82].

This evidence calls for* in vivo* and* in vitro* studies to verify the role of the oxidized, senescent RBCs or RBC-derived vesicles on macrophage polarization within haemorrhagic atherosclerotic lesions. The crosstalk between macrophages and RBCs might represent a potentially novel mechanism by which oxidative stress can contribute to atherosclerotic disease progression.

### 3.3. Crosstalk between Red Blood Cells and T Lymphocytes

The binding of circulating immune cells to the vascular wall is a central process in inflammation, metastasis, and therapeutic cell delivery.* In vivo* and* in vitro* studies show that RBCs facilitate the engagement of circulating lymphocytes within the vascular endothelium [15, 83]. It is well known that during inflammation and local immune response, capillary diameter and blood flow increase. This may allow the extravasation of RBCs, together with activated T cells, to the inflammatory place. A number of studies during the last three decades have presented evidence pointing to RBCs as putative modulators of T cell proliferation, both* in vitro* and* in vivo* [1]. In particular, RBCs are able to enhance T cell expansion and survival by inhibiting activation-induced T cell death, an effect possibly associated with a decrease of oxidative stress within activated T cells [14]. In previous studies optimal T cell proliferation and survival were only observed with intact RBCs and when RBCs were in close contact or proximity with activated T cells [14]. The need for RBC integrity and proximity might imply the involvement of interactions between receptors on each cell type, as suggested by some authors [84, 85]. Recently it has been demonstrated that RBCs release protein factors with the capacity to sustain T cell growth and survival [13]. Our group demonstrated that RBCs from patients with carotid atherosclerosis maintain the ability to sustain* in vitro* the proliferative response of activated T lymphocytes [17]. Our results suggest that structural alterations and redox imbalance in patient RBCs do not prevent them from releasing some protein factors promoting T cell proliferation. Proliferating T cells in our study produced high concentrations of IFN-*γ*, the Th1 cytokine involved in the inflammatory processes leading to plaque progression [86, 87]. In contrast to us some studies suggest that RBC-derived exosomes are potential modifiers of T cell responses in addition to their reported roles in innate immunity and B cell responses [88, 89]. The adverse patient outcomes associated with RBC storage length, observed in several clinical studies, well support our hypothesis on a pathogenic role of oxidized/senescent RBCs in complex inflammatory diseases such as atherosclerosis.

Stored RBCs, accompanied by shedding and release of extracellular vesicles, are capable of stimulating peripheral blood mononuclear cells (PBMC)* in vitro* and provoking proinflammatory cytokine response [41, 90]. Additionally, vesicle-derived RBCs augment mitogen-driven T cell proliferation in PBMC cultures in an antigen presenting cell-and cell contact-dependent manner [91, 92].

Apoptotic cell death has been demonstrated in advanced human atherosclerotic plaques [93] and impaired phagocytosis of apoptotic cells has been proposed as a mechanism that contributes to necrotic core formation to plaque vulnerability and rupture [94, 95]. Our research group has showed that unlike RBCs obtained from healthy subjects, RBCs from patients and* in vitro* oxidized RBCs did not protect activated T lymphocytes from apoptosis [17]. Hence, RBCs from patients with carotid atherosclerosis, probably due to their oxidative imbalance, impact T cell integrity and function.

## 4. Conclusion

Collectively, literature data demonstrate that RBCs represent not only an efficacious mechanism to counteract oxidative stress but also a further tool to maintain immunological homeostasis. However, when an intense production of reactive species takes place, RBCs may acquire a prooxidant behavior [18] and lose their typical structural and functional features [16–18, 96]. In particular, the oxidative injuries on RBC membrane, cytoskeleton, and cytoplasm components likely represent dangerous signals for the innate (e.g., DCs and macrophages) and adaptive immunity (e.g., T lymphocytes), thus contributing to or even triggering a damaging process worsening atherosclerotic disease. Improving our knowledge on the molecular basis of cellular interactions could offer opportunities for clinical intervention exploiting RBC and immune cell cooperation.
